# HIF-1-mediated suppression of mitochondria electron transport chain function confers resistance to lidocaine-induced cell death

**DOI:** 10.1038/s41598-017-03980-7

**Published:** 2017-06-19

**Authors:** Akihisa Okamoto, Chisato Sumi, Hiromasa Tanaka, Munenori Kusunoki, Teppei Iwai, Kenichiro Nishi, Yoshiyuki Matsuo, Hiroshi Harada, Keizo Takenaga, Hidemasa Bono, Kiichi Hirota

**Affiliations:** 1 Department of Anesthesiology, Kansai Medical University, Hirakata, Japan; 2 Department of Human Stress Response Science, Institute of Biomedical Science, Kansai Medical University, Hirakata, Japan; 3 Laboratory of Cancer Cell Biology, Radiation Biology Center, Kyoto University, Kyoto, Japan; 4 Precursory Research for Embryonic Science and Technology (PRESTO), Japan Science and Technology Agency (JST), Saitama, Japan; 5 Department of Life Science, Shimane University Faculty of Medicine, Izmo, Japan; 6 Database Center for Life Science (DBCLS), Research Organization of Information and Systems (ROIS), Mishima, Japan

## Abstract

The local anesthetic lidocaine induces cell death by altering reactive oxygen species (ROS) generation and mitochondrial electron transport chain function. Because hypoxia-inducible factor 1 (HIF-1) is involved in determining oxygen metabolism and mitochondria function, we investigated the involvement of HIF-1 activity in lidocaine-induced cell death. We investigated the role of HIF activation on lidocaine-induced caspase activation and cell death in renal cell-derived RCC4 cells lacking functional von Hippel-Lindau (VHL) protein. We demonstrate that HIF-1 suppressed oxygen consumption and facilitated glycolysis in a pyruvate dehydrogenase kinase-1-dependent manner and that activation of HIF-1 conferred resistance to lidocaine-induced cell death. We also demonstrated that exogenous HIF-1 activation, through HIFα-hydroxylase inhibition or exposure to hypoxic conditions, alleviates lidocaine toxicity by suppressing mitochondria function and generating ROS, not only in RCC4 cells, but also in the neuronal SH-SY5Y cells. In conclusion, we demonstrate that HIF-1 activation due to VHL deletion, treatment with small molecule HIFα-hydroxylase inhibitors, and exposure to hypoxic conditions suppresses mitochondrial respiratory chain function and confers resistance to lidocaine toxicity.

## Introduction

Local anesthetics, including lidocaine, affect the intra- and extra-cellular signaling pathways of both neuronal and non-neuronal cells, resulting in long-term modulation of biological functions such as cell growth and death^1^. Although the primary target of lidocaine is voltage-gated sodium channels, the targets and mechanisms in the context of cell growth and death are unknown. Studies indicate that mitochondria are one of the critical targets of lidocaine^2–4^. Similarly, we previously reported that reactive oxygen species (ROS) derived from mitochondria play an essential role in lidocaine-induced apoptosis and treatment with the antioxidants *N*-acetyl cysteine (NAC) and Trolox effectively prevents apoptosis^4^. The mitochondrial oxidative phosphorylation (OXPHOS) system plays a critical role in modulating ATP supply in the human body. Deficits derived from genetic and pharmacologic treatments cause functional disorders and cell death. The OXPHOS machinery consists of four enzyme complexes in the respiratory chain that transport electrons obtained from the oxidation of carbohydrates and fats to molecular oxygen (O_2_); a fifth enzyme complex uses the energy derived from this process to drive ATP synthesis. O_2_ is the electron acceptor, resulting in the production of H_2_O in a process that is catalyzed by complex IV of the mitochondrial electron transport chain (ETC). Because the process is not completely efficient, electron transport to O_2_ may occur in complexes I or III, resulting in generation of ROS that can oxidize cellular proteins, lipids, and nucleic acids^5–7^. The generated ROS causes cell dysfunction or death when cells are exposed to drugs affecting electron transport in the ETC.

The hypoxia-inducible factors (HIFs) HIF-1 and HIF-2 are transcriptional factors that function as a master regulators of oxygen homeostasis in most animal species^8–11^. Previous studies demonstrated that HIF-1 transactivates genes encoding glucose transporters, glycolytic enzymes, and the pyruvate dehydrogenase kinase PDK-1 in response to reduced O_2_ availability^12, 13^. Each HIF is a heterodimeric protein composed of a constitutively expressed HIF-1β subunit and an O_2_-regulated HIF-1α or HIF-2α subunit^14^. Under aerobic or normoxic conditions, HIF-1α and HIF-2α are subjected to prolyl hydroxylation by oxygenases that utilize O_2_ as a substrate^9^.

Hydroxylation modification is required for binding of the von Hippel-Lindau (VHL) protein, which targets HIF-1α and HIF-2α for ubiquitination and proteasomal degradation^15^. The interaction of HIFα with the coactivators CBP and p300 is blocked by O_2_-dependent asparaginyl hydroxylation mediated by the factor inhibiting HIF-1 (FIH-1) protein^16, 17^. Under hypoxic conditions, the hydroxylation reaction rate declines because of a shortage in substrate O_2_ molecules leading to HIF activation and providing a mechanism by which changes in oxygenation are transduced to the nucleus as changes in gene expression. Even under normoxic conditions, treatment with hydroxylase inhibitors such as dimethyloxaloylglycine (DMOG), desferoxamine (DFX), and n-propyl gallate (nPG) activate HIFs^14, 18, 19^. Moreover, certain types of mutations and the genetic deletion of VHL also activate HIFs even under normoxic conditions^18, 20^.

HIFs drive hypoxic gene expression changes that are thought to be adaptive for cells exposed to a reduced-oxygen environment. Mitochondrial function can be regulated by HIFs^12, 13, 21, 22^. OXPHOS is regulated by several mechanisms, including substrate availability. The major substrates for OXPHOS are O_2_, which is the terminal electron acceptor, and pyruvate, which is the primary carbon source. Pyruvate is the end product of glycolysis and is converted to acetyl-CoA through the activity of the pyruvate dehydrogenase complex of enzymes that is regulated by pyruvate dehydrogenase kinases (PDKs)^12^. The acetyl-CoA directly enters the tricarboxylic acid cycle (TCA) cycle as citrate synthase where it combines with oxaloacetate to generate citrate. Thus, HIF-1 not only regulates the supply of oxygen, but also actively regulates the oxygen demand of the tissues and cells by reducing the activity of the major cellular consumer of oxygen, mitochondria^22^. HIF-1 promotes the efficient use of available oxygen while also reducing the generation of harmful byproducts from respiration such as free radicals and H_2_O_2_^23, 24^.

In this study, we investigate the role of HIF activation on lidocaine-induced apoptosis in renal cell-derived RCC4 cells lacking functional VHL and neuronal SH-SY5Y cells exposed to nPG, DFX, or hypoxia and describe that activation of HIF-1 alleviates lidocaine toxicity by suppressing mitochondrial functions and generating ROS.

## Results

### RCC4-EV cells are more resistant to lidocaine-induced cell injury than RCC4-VHL cells

**Figure 1:**
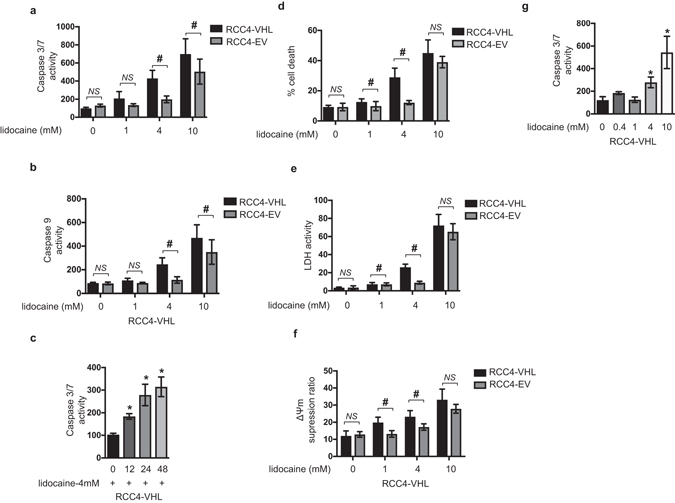
RCC4-EV cells are more resistant to lidocaine-induced cell injury than RCC4-VHL cells. RCC4-VHL and RCC4-EV cells were exposed to the indicated concentrations (1, 4, or 10 mM) of lidocaine for varying lengths of time (0, 12, 24, and 48 h). (**a**,**b** and **c**) Graphic depictions of caspase-3/7 (n = 5) (**a** and **b**) and caspase-9 (n = 5) (**c**) activity in each treatment group at different time points (**d**) Cells were harvested and cell death percentages were measured with flow cytometry. The ratio of propidium iodide (PI)-positive and/or annexin V-positive cells [(Q1 + Q2 + Q4)/(Q1 + Q2 + Q3 + Q4)] was used to calculate the percentage of dead cells (Supplemental Fig. S1) (n = 3). (**e**) Graphic depiction of cell death percentages in treated and untreated cell populations. Cell death was evaluated by measuring the levels of lactate dehydrogenase (LDH) within culture supernatants (n = 4). Control is treatment with lysis buffer. (**f**) Graphic depiction of the average mitochondrial membrane potential (ΔΨm) of treated and untreated cells (n = 3) at each time point. Values indicate the ratio [Q2/(Q2 + Q4)] of green JC-1 monomers (527 nm emission) to red aggregates (590 nm emission). Data presented in (**a**–**f**) are expressed as mean ± standard deviation (SD). Differences between results were evaluated by two-way ANOVA followed by Dunnett’s test for multiple comparisons (**a**, **c**–**f**) or one-way ANOVA followed by Dunnett’s test for multiple comparisons (**b**). *p < 0.05 compared to the control cell population at incubation time 0 h (no treatment). ^#^ *p* < 0.05 compared to the control cell population at the same lidocaine concentration (group).

RCC4-EV cells lack VHL activity and constitutively express HIF-1α and HIF-2α proteins, whereas in the RCC4-VHL subclone, which is stably transfected with a VHL expression vector, the expression of HIF-1α and HIF-2α proteins are induced only under hypoxic culture conditions^20, 25^. In order to determine the dose-response relationship for caspase 3/7 activation, we treated RCC4-VHL cells with the indicated doses of lidocaine (Supplementary Fig. 1a). Both RCC4-VHL and RCC4-EV cells were exposed to 1, 4, and 10 mM lidocaine for 12 h and the caspase 3/7 (Fig. 1a) and caspase 9 (Fig. 1b) activity was evaluated. Next, time-response relationship was detemined for the indicated times (Fig. 1c). Concentrations higher than 4 mM lidocaine induced caspase activation within 12 h, and 4 mM lidocaine induced the activation in a time-dependent manner up to 48 h.

A significant difference was detected between RCC4-EV cells and RCC4-VHL cells following 4 mM and 10 mM lidocaine treatment. To confirm these findings, RCC4-EV cells and RCC4-VHL cells were stained with PI and FITC-conjugated annexin V and evaluated by flow cytometry (Fig. 1d and Supplementary Fig. 1b). In agreement with the activation of caspase 3/7 and caspase 9, cell death rates following treatment with 1 mM and 4 mM lidocaine for 12 h were significantly less in RCC4-EV cells than in RCC4-VHL cells. The 10 mM lidocaine treatment increased the PI positive cell population, whereas the component ratios were not different between the cell types (Fig. 1d). Although there was a significant difference between RCC4-VHL cells and RCC4-EV cells in the lactate dehydrogenase (LDH) release induced by 4 mM lidocaine treatment, a significant difference was not detected following treatment with 10 mM lidocaine (Fig. 1e).

Finally, the mitochondria membrane voltage (ΔΨm) was investigated in both cell lines (Fig. 1f and Supplementary Fig. 1c). The decrease in ΔΨm by 1 mM and 4 mM lidocaine treatment was prevented in RCC4-EV cells. In contrast, a statistically significant difference was not detected following 10 mM lidocaine treatment.

Together, the experimental results indicate that RCC4-EV cells are more resistant to 4 mM lidocaine-induced apoptosis compared to its derivative RCC4-VHL, in which VHL protein are exogenously overexpressed.

### HIFs are activated in RCC4-EV cells under 20% O_2_ conditions

**Figure 2:**
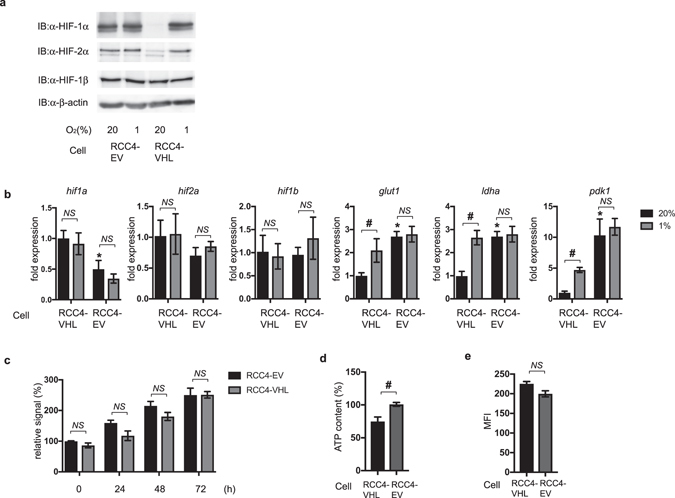
HIFs are activated in RCC4-EV cells in normoxic conditions. (**a**) RCC4-EV and RCC4-VHL cells were exposed to normoxic (20% O_2_) or hypoxic (1% O_2_) conditions for 4 h. Whole-cell lysates were immunoblotted (IB) using anti-HIF-1α, HIF-2α, HIF-1β, and β-actin antibodies. Experiments were repeated twice and representative blots are shown. (**b**) RCC4-EV and RCC4-EV cells were cultured for 4 h under 20% O_2_ or 1% O_2_ conditions prior to analysis of hypoxia-inducible factor 1α (hif1α), hypoxia-inducible factor 2α (hif2α), hypoxia-inducible factor 1β (hif1β), glucose transporter 1 (glut1), lactate dehydrogenase A (lhda), and pyruvate dehydrogenase kinase 1 (pdk1) mRNA levels using real-time reverse transcriptase polymerase chain reaction (RT-PCR). Fold expression was calculated relative to the values measured for RCC4-VHL cells incubated in 20% O_2_. Data presented are expressed as mean ± standard deviation (SD). *p < 0.05 compared to the control cell population of RCC4-VHL cells under 20% O_2_ conditons. ^#^ *p* < 0.05 compared between indicated groups. (**c**) Graphic depiction of the cell viability levels for treated and untreated cells at each time point (n = 4). Differences between results were evaluated by two-way ANOVA followed by Dunnett’s test for multiple comparisons. (**d**) ATP content of RCC4-EV and RCC4-VHL cells was measured. Differences between results were evaluated by *t*-test. ^#^ *p* < 0.05 compared between indicated groups. (**e**) Equal numbers of RCC4 and RCC4-VHL cells were stained with MitoGreen^TM^ and analyzed by flow cytometry to measure mitochondrial mass. Differences between results were evaluated by *t*-test.

We investigated whether the HIFs are active in RCC4-VHL and RCC4-EV cells in normoxic (20% O_2_) and hypoxic (1% O_2_) conditions. First, expression of HIF-1α, HIF-2α, and HIF-1β proteins were assayed by Western blot analysis in RCC4-VHL cells and RCC4-EV cells under both 20% and 1% O_2_ conditions (Fig. 2a). Consistent with a previous report^20^, HIF-1α and HIF-2α proteins were constitutively expressed even under 20% O_2_ conditions in RCC4-EV cells at comparable levels to those of RCC4-VHL cells under 1% O_2_ conditions. HIF-1β expression was stable for both oxygenation states in both cell types. Consistent with HIF-α protein expression, glucose transporter 1 (glut1), lactate dehydrogenase A (ldha), and PDK-1 mRNA had increased expression in RCC4-EV cells than in RCC4-VHL cells under 20% O_2_ conditions. In contrast, HIF-1α and HIF-2α mRNA expression patterns are different from those of HIF-downstream gene expressions (Fig. 2b). Notably, RCC4-EV cells under 20% O_2_ conditions had decreased HIF-1α mRNA expression levels compared to RCC4-VHL cells under 1% O_2_ conditions (Fig. 2b).

Next, the RCC4-EV and RCC4-VHL cell proliferation rates were measured using an assay based on reduction of the tetrazolium compound [3-(4,5-dimethyl-2-yl)-5-(3-carboxymethoxyphenyl)-2-(4-sulfophenyl)-2H-tetrazolium, inner salt; MTS] (Fig. 2c). No significant difference in cell proliferation rates between RCC4-VHL cells and RCC4-EV cells was found. However, the ATP content in RCC4-EV cells was higher than that in RCC4-VHL cells (Fig. 2d). There were no significant differences in mitochondrial mass between RCC4-VHL cells and RCC4-EV cells (Fig. 2e).

The transcription factors HIF-1 and HIF-2 were constitutively activated in RCC4-EV, which lacks VHL gene even under 20% O_2_ as comparably as those of RCC4-VHL cells under 1% O_2_ conditions.

### HIF-1 activation is required for RCC4-EV cells to confer resistance to lidocaine-induced cell death

**Figure 3:**
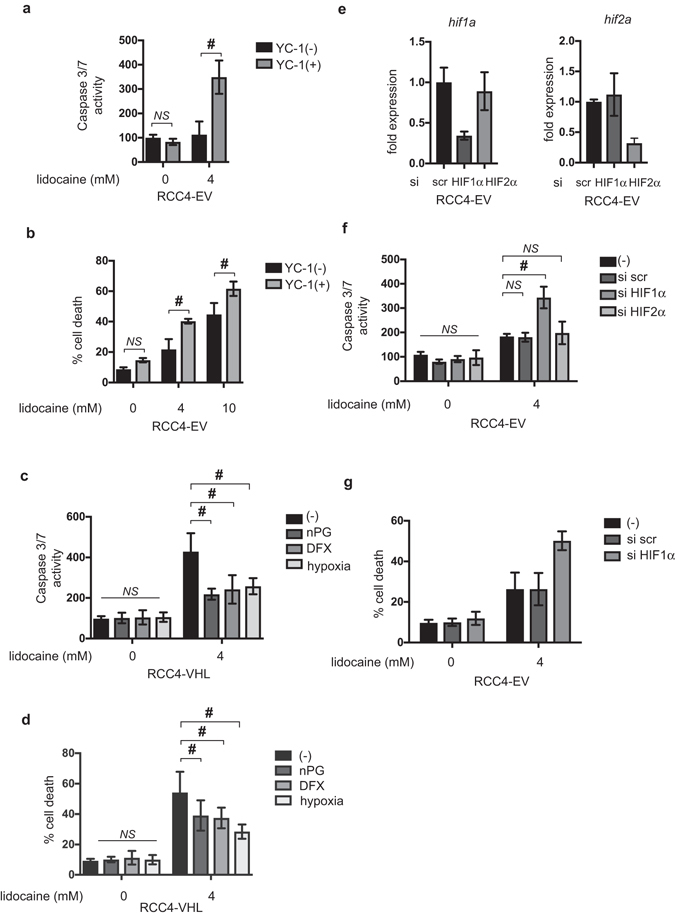
HIF-1 activation is required for RCC4-EV cells to confer resistance against lidocaine-induced cell death. (**a** and **b**) RCC4-EV cells were exposed to 4 mM lidocaine for 24 h with or without 100 µM YC-1. (**a**) Graphic depictions of caspase-3/7 activity (n = 5). (**b**) Cells were harvested and cell death percentages were analyzed by flow cytometry. (**c** and **d**) RCC4-VHL cells were exposed to 4 mM lidocaine and treated with 100 µM nPG, 100 µM DFX, or exposure to 1% O_2_ conditions for 24 h. Graphic depictions of caspase-3/7 activity (n = 5). (**c**) Cells were harvested and cell death percentages were analyzed by flow cytometry. (**d**) (**e**–**g**) RCC4-EV cells were transfected with small interfering RNA (siRNA) targeting HIF-1α (hif1a), HIF-2α (hif2a), or a negative control (scr). Expression of mRNA of HIF-1α, HIF-2α was measued by *q*RT-PCR. (**e**) Cells were exposed to 4 mM lidocaine for 24 h. Graphic depictions of caspase-3/7 activity (n = 5). (**f**) Cells were harvested and cell death percentages were analyzed by flow cytometry. (**g**) Data presented in (**a**–**g**) are expressed as mean ± standard deviation (SD). Differences between results were evaluated by two-way ANOVA followed by Dunnett’s test for multiple comparisons. *p < 0.05 compared to the control cell population at incubation time 0 h (no treatment). ^#^ *p* < 0.05 compared to the indicated experimental group.

HIFs, including HIF-1 and HIF-2, are activated in RCC4-EV cells under 20% O_2_ conditions. We next examined the involvement of HIFs in maintaining the resistance to lidocaine toxicity. RCC4-EV cells were treated with the HIF inhibitor YC-1. The YC-1 treatment decreased expression of genes located downstream from HIF-1 and HIF-2 (Supplementary Fig. 2a). The YC-1 treatment significantly increased caspase3/7 activation (Fig. 3a) and induced cell death (Fig. 3b) within 12 h following 4 mM lidocaine treatment in RCC4-EV cells. In contrast, the treatment with YC-1 did not exert a significant effect on caspase3/7 activation or cell death rates in RCC4-VHL cells (Supplementary Fig. 2b). Next, we treated RCC4-VHL cells with the HIFα-hydroxylase inhibitors nPG (100 µM) and DFX (130 µM) in addition to exposure to the 1% O_2_ condition. These treatments increased expression of genes located downstream of HIF-1 and HIF-2 (Supplementary Fig. 2c) and conferred lidocaine resistance in RCC4-VHL cells. The treatments significantly suppressed caspase3/7 activation (Fig. 3c) and inhibited cell death (Fig. 3d) induced by 4 mM lidocaine in RCC4 cells. Thus, the evidence indicates that HIF activation is required and sufficient for maintenance of resistance against lidocaine toxicity. Because genetic ablation or treatment with HIFα hydroxylase inhibitors including nPG and DFX activate both HIF-1 and HIF-2 transcription factors^19, 20^, we examined which subtype is critical for conferring lidocaine resistance. Both the mRNA and protein expressions of HIF-1α or HIF-2α was were decreased decerased significantly by treatment treament with the corresponding siRNA (Fig. 3e) and HIFα proteins (Supplementary Fig. 2d). The knockdown study indicated that suppression of HIF-1α expression, but not HIF-2α, significantly affected caspase 3/7 activation (Fig. 3f) and cell death rates (Fig. 3g) following 4 mM lidocaine treatment. The results indicate that HIF-1, but not HIF-2, plays an essential role in the phenomenon.

Thus, HIF-1 but not HIF-2 activity is required for RCC4-EV cells to be resistant to lidocaine-induced cell death.

### Reprogrammed oxygen metabolism is established in RCC4-EV cells

**Figure 4:**
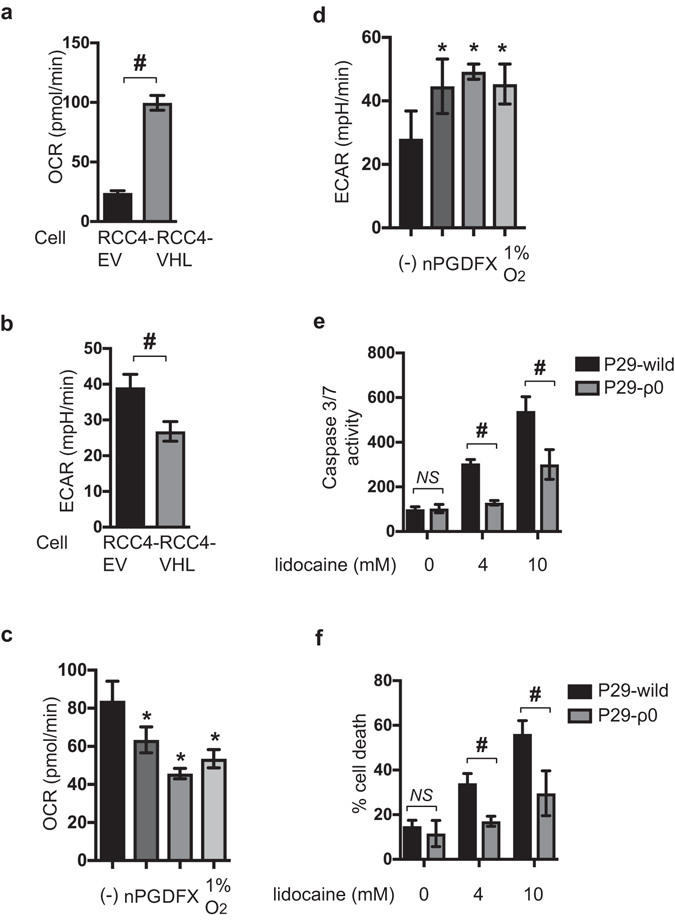
Reprogrammed oxygen metabolism is established in RCC4-EV cells. Oxygen consumption rate (OCR) (**a** and **c**) and extracellular acidification rate (ECAR) (**b** and **d**) in RCC4-EV cells and RCC4-VHL cells (**a** and **b**) in normoxic (20% O_2_) conditions and RCC4-VHL cells exposed to nPG, DFX, or hypoxic (1% O_2_) conditions (**c** and **d**). P29 cells and P29 ρ0 cells were exposed to the indicated lidocaine dose for 24 h (**e** and **f**). Graphic depiction of caspase-3/7 activity (n = 5) (**e**). Cells were harvested, and the levels of cell death percentages were analyzed by flow cytometry. The ratio of propidium iodide (PI)-positive and/or annexin V-positive cells [(Q1 + Q2 + Q4)/(Q1 + Q2 + Q3 + Q4)] was used to calculate the percentage of dead cells (Supplemental Fig. S1) (n = 3) (**f**). Cells were harvested, and the levels of cell death were analyzed by flow cytometry (f). The ratio of propidium iodide (PI)-positive and/or annexin V-positive cells [(Q1 + Q2 + Q4)/(Q1 + Q2 + Q3 + Q4)] was used to calculate the percentage of dead cells (n = 3). Data presented in (**a**–**f**) are expressed as means ± standard deviations (SD). Differences between results were evaluated by the *t*-test (**a** and **b**), one-way ANOVA (**c** and **d**) followed by Dunnett’s test for multiple comparisons (**c** and **d**), and two-way ANOVA (**e** and **f**) followed by Dunnett’s test for multiple comparisons (**e** and **f**). *p < 0.05 compared to the control cell population. ^#^ *p* < 0.05 compared to the indicated experimental groups.

Because numerous reports clearly indicate HIF-1 involvement in determination of oxygen metabolism^12, 13, 21, 24, 26^, we investigated gene expression in RCC4-EV cells and RCC4-VHL cells by RNA-Seq analysis using data deposited in a public database. The RNA expression analysis by Next-Generation Sequencing (NGS) data revealed differences in the cellular hypoxic pathway and HIF-1-signaling pathway between RCC4-EV cells and RCC4-VHL cells (Supplementary Fig. 3a and b and Supplementary Table 1). Next, we investigated oxygen metabolism activity and glycolysis in RCC4-EV cells and RCC4-VHL cells using the oxygen consumption rate (OCR) (Fig. 4a and Supplementary Fig. 4a and c) and extracellular acidification rate (ECAR), which is a surrogate index for glycolysis (Fig. 4b and Supplementary Fig. 4b and d). The mitochondrial basal oxygen consumption rate (OCR-basal) was significantly lower in RCC4-EV cells than in RCC4-VHL cells under 20% O_2_ conditions (Fig. 4a). In accordance, ECAR levels, representing extracellular lactate concentration, were significantly higher in RCC4-EV cells than in RCC4-VHL cells (Fig. 4b). In addition, there were significant differences in maximum respiratory rate, non-mitochondrial respiration, and proton leak between RCC4-EV cells and RCC4-VHL cells (Supplementary Fig. 4e–g) and the reprogramming is suppressed by YC-1 treatment (Supplementary Fig. 5a). Thus, oxygen and metabolic reprogramming from aerobic to anaerobic glucose metabolism occurred in RCC4-EV cells. Moreover, nPG treatment, DFX treatment, or 1% O_2_ exposure conditions also metabolically reprogrammed RCC4-VHL-cells (Fig. 4c and d). Next, the involvement of mitochondria function in lidocaine-induced toxicity was investigated. P29 cells are an established cell line derived from mouse lung cancer. P29 ρ0 cells are a subclone lacking mitochondria DNA (Supplementary Fig. 5b). Both 4 and 10 mM lidocaine induced caspase 3/7 activation (Fig. 4e) and cell death rates (Fig. 4f) assayed using FACS in P29 cells, similar to RCC4-VHL cells. In contrast, P29 ρ0 cells are almost resistant to the effects induced by 4 and 10 mM lidocaine (Fig. 4e and f).

The experimental results indicated the HIF-1 activation is required and sufficient to reprogram the oxygen metabolism in a mitochondria-dependent manner in RCC4 cells.

### ROS production in response to lidocaine treatment is reduced in RCC4-EV cells in a PDK-1-dependent manner

**Figure 5:**
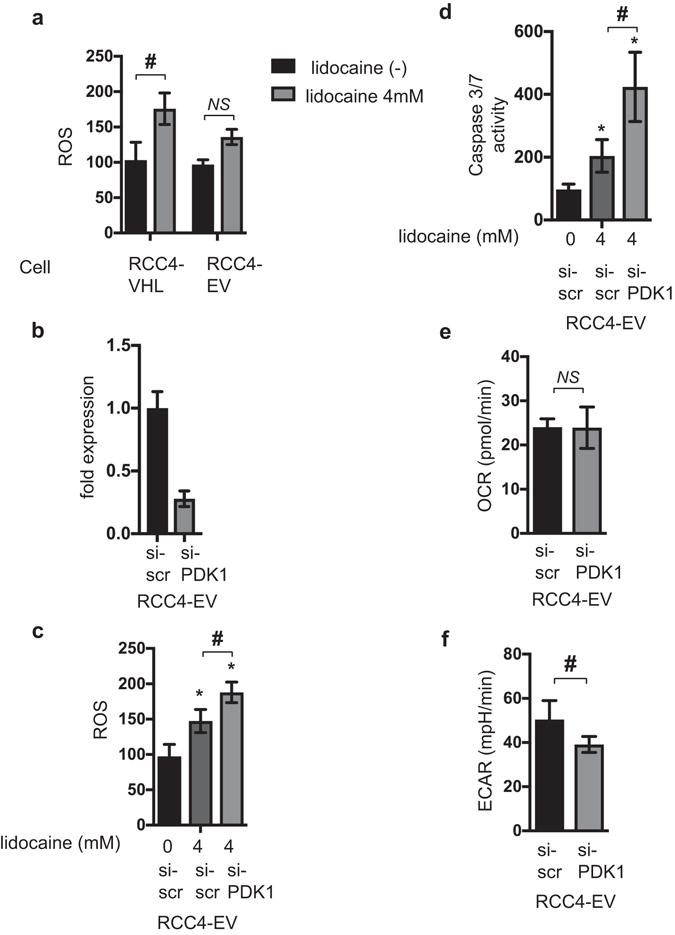
ROS production in response to lidocaine treatment is reduced in RCC4-EV cells in a PDK-1-dependent manner. (**a**) Graphic depiction of reactive oxygen species (ROS) production in RCC4-VHL cells and RCC4-EV cells exposed to 4 mM lidocaine (n = 3). Data depict the ratio of ROS production in treated cells to compared to that in the mean value of RCC4-VHL cells without lidocaine treatment. RCC4-EV cells were transfected with small interfering RNA (siRNA) targeting pyruvate dehydrogenase kinases 1 (PDK-1) or a negative control (scr). (**c**) ROS production during treatment with 4 mM lidocaine was assayed. (**d**) Graphic depictions of caspase-3/7 activity (n = 5). The cellular oxygen consumption rate (OCR) (**e**) and extracellular acidification rate (ECAR) (**f**) were measured. Data presented in (**a**–**f**) are expressed as mean ± standard deviation (SD). Statistical differences between results were evaluated using the *t*-test (**a**,**d** and **e**) or two-way ANOVA followed by Dunnett’s test for multiple comparisons (**b**–**d**). *p < 0.05 compared to the control cell population. ^#^ *p* < 0.05 compared to the indicated experimental groups.

In a previous study^4^, we found that lidocaine induces reactive oxygen species (ROS) generation in cells and that ROS play a critical role in lidocaine-induced cell death. Here, we compared ROS generation between RCC4-VHL and RCC4-EV cells in response to 4 mM lidocaine treatment. ROS generation in response to 4 mM lidocaine treatment increased in RCC4-VHL cells, but did not significantly change in RCC4-EV cells (Fig. 5a). PDK-1 expression is responsible for the reduction in ROS generation under hypoxic conditions^23, 24^ and the metabolic switch for cellular adaptation to hypoxia^12, 13^. mRNA expression of PDK-1 was decreased significantly by treatment with the siRNA (Fig. 5b). The reduction of ROS generation in RCC4-EV cells was reversed by suppression of PDK-1 expression by treatment with siRNA against PDK-1 (Fig. 5c). Accordingly, caspase 3/7 activation by 4 mM lidocaine is increased in RCC4-EV cells treated with siRNA against PDK-1 (Fig. 5d). Although the OCRs were not significantly affected by treatment with siRNA against PDK-1 (Fig. 5e), ECAR in RCC4-EV cells was suppressed (Fig. 5f).

Thus, ROS production in response to lidocaine treatment is reduced in RCC4-EV cells in a PDK-1-dependent manner. Suppression of ROS generation results in survival of cells against lidocaine treatment.

### HIF-1 activation is sufficient to resist lidocaine toxicity in neuronal SH-SY5Y cells

**Figure 6:**
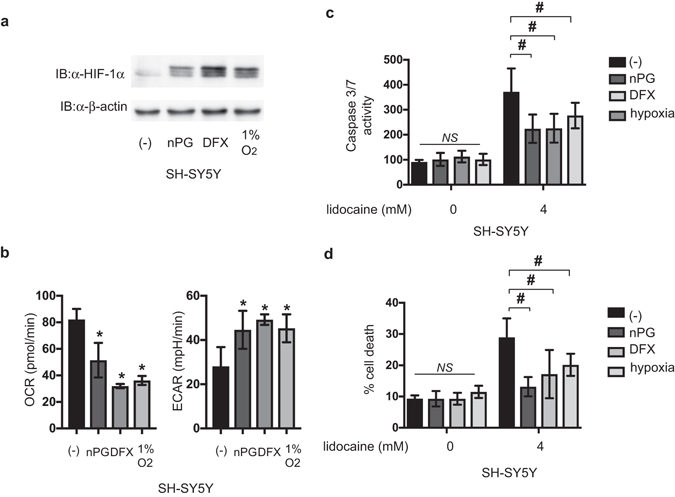
HIF-1 activation is sufficient to resist lidocaine toxicity in neuronal SH-SY5Y cells. (**a**) SH-SY5Y cells were exposed to 100 µM nPG, 100 µM DFX, or 1% O_2_ for 4 h. Whole-cell lysates were immunoblotted (IB) using anti-HIF-1α, HIF-1β, or β-actin antibodies. Experiments were repeated twice and representative blots are shown. (**b**) Oxygen consumption rate (OCR) and extracellular acidification rate (ECAR) of SH-SY5Y cells exposed to 100 µM nPG, 100 µM DFX, or 1% O_2_ for 6 h were measured. (**c**) SH-SY5Y cells were exposed to 4 mM lidocaine and 100 µM nPG, 100 µM DFX, or 1% O_2_ for 24 h. Graphic depictions of caspase-3/7 activity (n = 5). (**d**) SH-SY5Y cells were exposed to 4 mM lidocaine and 100 µM nPG, 100 µM DFX, or 1% O_2_ for 24 h. Cells were harvested and cell death percentages were analyzed by flow cytometry (n = 3). *p < 0.05 compared to the control cell population. ^#^ *p* < 0.05 compared to the indicated experimental groups.

Finally, we tested whether exogenous HIF-1 activation confers resistance to lidocaine in cells derived from different origins than RCC4 cells. The neuronal SH-SY5Y cells were treated with 100 µM nPG or 130 µM DFX in 20% O_2_ or exposed to 1% O_2_ for 6 h. The treatment increased HIF-1α protein expression (Fig. 6a), suppressed OCR, and increased ECAR (Fig. 6b) in SH-SY5Y cells and induced expression of HIF-1α-target genes (Supplementary Figure 6). As in RCC4-VHL cells, treatment with nPG or DFX conferred resistance to 4 mM lidocaine-induced caspase 3/7 activation (Fig. 6c) and cell death rates assayed with PI/Annexin V staining (Fig. 6d). In addition to the treatment with nPG and DFX, exposure to 1% O_2_ also conferred lidocaine resistance to SH-SY5Y cells (Fig. 6c and d).

Thus, HIF-1 activation by treatment with HIFα-hydroxylase inhibitors and exposure to hypoxia also sufficient to be resistant against lidocaine toxicity to cells even derived from neuronal origin.

## Discussion

In this study, we demonstrated for the first time that activation of the endogenous transcription factor HIF-1 attenuated ROS generation and cell death elicited by lidocaine in not only RCC4 cells, the established renal carcinoma cell line, but also SH-SY5Y cells, a neuronal cell line.

Although the primary target of lidocaine as a local anesthetic is the voltage-gated sodium channels^1^, lidocaine affects cell growth and cell death by modulating mitochondria function at clinically relevant concentrations. Our experimental results utilizing P29 ρ0 cells that lack mitochondria DNA (Fig. 4e and f) clearly indicate mitochondria play a critical role in lidocaine-induced apoptosis but not necrosis^2–4^. We recently demonstrated that the local anesthetic lidocaine suppresses the mitochondrial ETC in neuronal SH-SY5Y cells in a dose- and time-dependent manner, thereby attenuating mitochondrial membrane potential, inducing ROS production, and activating caspase-9- and caspase-3/7-mediated apoptosis and necrosis^4^. Genetic deletion of VHL gene results in activation of HIFs, including HIF-1 and HIF-2, as demonstrated in Fig. 2a and Supplementary Figure 3. VHL is an essential component of the E3 ubiquitin ligase that plays an essential role in regulation of HIFα subunit protein expression in response to ambient oxygen concentrations. Thus, in RCC4-EV cells, HIF-1α and HIF-2α proteins are expressed even under 20% oxygen concentrations^20^. VHL deletion conferred resistance against 1-4 mM lidocaine-induced apoptosis and overexpression of VHL in RCC4 cells made cells sensitive to lidocaine toxicity (Figs 1 and 3). Although both HIFs are structurally and functionally similar, HIF-1 and HIF-2 exert distinct roles^27, 28^. In Fig. 3f, the differential suppression of f HIF-1α and HIF-2α mRNA with siRNA indicate that HIF-1, rather than HIF-2, plays a dominant and critical role in conferring the resistance to lidocaine-induced apoptosis. This is consistent with the reports that HIF-1 is preferentially involved in metabolic regulation, especially glucose metabolism^12, 13, 29, 30^. Both RT-PCR assay (Fig. 2b) and RNA-Seq analysis (Supplementary Figure 3b) reveal that expression of HIF-1α mRNA is statistically significantly suppressed in RCC4-EV cells compared to RCC4-VHL cells. Although the exact molecular mechanism behind the phenomenon is to be investigated, this may be due to the negative feed back loop.

The primary purpose of OXPHOS is the transfer of electrons through a series of cytochromes in order to make a proton gradient across the inner mitochondrial membrane. The potential energy of the proton gradient is utilized to synthesize ATP by ATP synthases. O_2_ is the electron acceptor, resulting in the production of H_2_O in a process that is catalyzed by cytochrome *c* oxidase (COX; complex IV). COX4 has two isoforms: COX4I1 and COX4I2. HIF-1 upregulates COX4I2 expression and activates the LON mitochondrial protease, which in turn degrades COX4I1^21^. This mechanism is part of the molecular machinery for preserving ATP production in RCC4-EV cells. In accordance with the evidence, the basal OCR of RCC4-EV is lower than that of RCC4-VHL (Fig. 4a). In addition, the Carbonyl cyanide 4-(trifluoromethoxy) phenylhydrazone (FCCP)-stimulated maximal respiration rates in RCC4-EV cells are decreased less than in RCC4-VHL cells (Supplementary Fig. 4e). Together, the evidence strongly suggests that the mitochondrial ETC in RCC4-EV cells is significantly inhibited compared to in RCC4-VHL cells. However, the mitochondrial mass and the mitochondrial membrane potential are equivalent in each cell line (Fig. 2e).

The ATP content was higher in RCC4-EV cells than in RCC4-VHL cells (Fig. 2d). Thus, as demonstrated by the significant difference in ECAR between RCC4-EV and RCC4-VHL cells, glycolysis in RCC4-EV cells is elevated to compensate for the suppression of OXPHOS. ATP production efficiency in RCC4-EV cells, defined as a decrease in OCR after treatment with the complex V inhibitor oligomycin, is lower than that in RCC4-VHL cells (Fig. 2e). Proton leak, as defined by the mitochondrial respiration rate in the presence of oligomycin, is apparent in RCC4-EV and RCC4-VHL cells (Supplementary Fig. 4g). Since mitochondrial superoxide production is steeply dependent on Δp, proton leak pathways may exist to minimize oxidative damage by tempering Δp and mitochondrial superoxide production^31–33^.

OXPHOS is regulated by several mechanisms, including substrate availability. The major substrate for OXPHOS is O_2_. Pyruvate is the product of glycolysis and is converted to acetyl-CoA through the activity of the pyruvate dehydrogenase complex of enzymes. Acetyl-CoA is another OXPHOS regulating factor. Acetyl-CoA directly enters the TCA cycle. The conversion of pyruvate to acetyl-CoA represents a critical regulatory point in cellular energy metabolism^34^. Pyruvate dehydrogenase is regulated by PDK phosphorylation of its E1 subunit^35, 36^. PDK1 is a HIF-1 downstream product that negatively regulates the function of the mitochondria by reducing pyruvate entry into the TCA cycle. By excluding pyruvate from mitochondrial consumption, PDK1 induction may increase the conversion of pyruvate to lactate, which is then shunted to the extracellular space, regenerating NAD for continued glycolysis. Several reports have also suggested a link between altered mitochondrial function in hypoxia and HIF activation^5–7^. Thus, HIF target gene activation is upstream of mitochondrial function, and responsible for altering mitochondrial activity in RCC4-EV cells^12, 13, 22^. The transcription factors HIF-1 and HIF-2 are identified to regulatory factors for a line of genes involving in intracellular metabolic regulation such as glycolysis and mitochondrial metabolism. In fact, a series of glycolytic enzyme such as glut1 and enzymes in TCA cycle such as isocitrate dehydrogenase 2 (IDH2) are reported to be induced under hypoxic conditions in a HIF-1-dependent manner in human umbilical vein endothelial cells. However, as indicated in our RNA-Seq analysis revealed that mRNA expression of IDH1, IDH2 or IDH3 was not significantly different between RCC4-EV cells and RCC4-VHL cells (gene_exp. diff, Supplementary Dataset S1). The evidence strongly suggests that these enzymes do not play a critical role in metabolic reprogramming and cell resistance to lidocaine-induced apoptosis. In contrast, expression of a line of glycolysis-related proteins including glut1 increases in RCC4-EV cells compared to RCC4-VHL cells.”

Previous reports and our recent findings indicate that both lidocaine-induced apoptosis and necrosis are ROS-dependent^37^. We recently demonstrated that lidocaine treatment induced ROS generation^4^. As such, these data imply that lidocaine-mediated cell death is dependent on mitochondrial function. Consistent with this conclusion, the mitochondrial DNA-deficient ρ0 cells were resistant to lidocaine treatment (Fig. 4e and f). O_2_ is the electron acceptor, resulting in the production of H_2_O in a process that is catalyzed by complex IV. Because the process is not completely efficient, electron transfer to O_2_ may occur at complex I or III, resulting in ROS generation, which oxidizes cellular proteins, lipids, and nucleic acids^5, 6, 38^. Lidocaine-induced ROS production in RCC4-EV cells is significantly less than in RCC4-VHL cells. Thus, HIF target gene activation is upstream of mitochondrial function and is responsible for altering mitochondrial activity. ROS generation in cancer cells occurs in a HIF-1-mediated metabolic reprogramming-dependent manner^39^.

In addition to regulation of HIF activity by VHL molecules, small chemical compounds activate HIFs by interfering the cellular HIFα hydroxylase systems^9^. We used two small chemical compounds, nPG competitive inhibition of 2-oxoglutarate^19^ and DFX essential cofactor Fe^2+^ chelate^9, 14^. nPG and DFX induced HIF-1α protein expression within 3 h (Fig. 6a). In accordance with the induction of HIF-1 activation, OCR decreased and ECAR increased within 6 h. The evidence indicates that not only continuous activation, but also transient activation of HIF-1 is sufficient to exert the metabolic reprogramming from OXPHOS to glycolysis and the reprogramming and mitochondria suppression are sufficient to prevent lidocaine-induced cell apoptosis. We also demonstrate that not only RCC4 cells derived from clear cell kidney carcinoma, but also the neuronal SH-SY5Y cells can acquire the lidocaine toxicity resistance.

**Figure 7:**
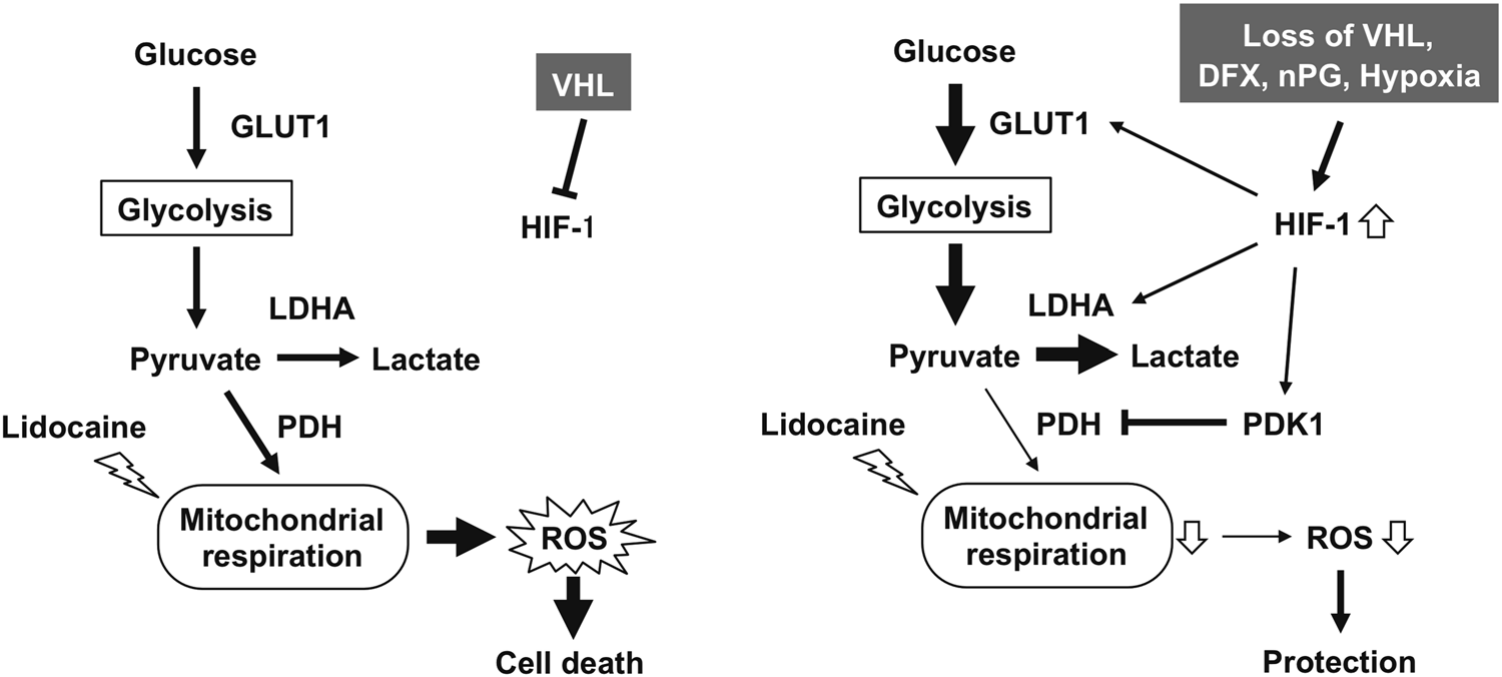
The schematic representation of the findings of this study. HIF-1α- induced PDK1 sequesters glucose metabolite flux from the mitochondria to glycolysis. This glycolytic shift redirects the metabolic flux to compensate the suppression of mitochondrial activity by lidocaine to reduce ROS generation and maintain intracellular ATP contents. LDHA: lactate dehydrogenase A, PDH: pyruvate dehydrogenase, PDK1: pyruvate dehydrogenase kinase, ROS: reactive oxygen species.

Reduced oxygen availability elicited by hypoxia and mitochondrial dysfunction leads to increased production of ROS by the electron transport chain. As demonstrated in this study, HIF-1 mediates adaptive metabolic responses to electron transport chain, including increased flux through the glycolytic pathway and decreased flux through the tricarboxylic acid cycle, in order to decrease mitochondrial ROS production (Fig. 7). HIF‐1 is also involved in mitochondrial one‐carbon (folate cycle) metabolism to increase mitochondrial antioxidant production (NADPH and glutathione). Thus, dynamic maintenance of ROS homeostasis is dependent on HIF-1. Recently, it was reported that VHL-deletion rescues the retardation of cell growth elicited by antimycin, a complex III inhibitor in the mitochondrial respiratory chain^40^. Interestingly, HIF-1 activation is necessary and sufficient for the phenomenon. In addition to the established cell lines, HIF-1 activation by chemical HIFα-hydroxylase inhibitor and exposure to hypoxic environments allows survival in zebrafish treated by antimycin and in a mouse model of Leigh syndrome harboring deletion of *Ndufs4*, a structural subunit in the mitochondrial complex I^41^.

In conclusion, we demonstrate that HIF-1 activation due to VHL deletion, treatment with small molecule HIFα-hydroxylase inhibitors, and exposure to hypoxic conditions suppresses mitochondrial respiratory chain function and confers resistance to lidocaine toxicity (Fig. 7).

## Methods

### Cell culture and reagents

The renal cell carcinoma cell lines stably transfected with pcDNA3-VHL (RCC4-VHL) or empty vector (RCC4-EV) were maintained in DMEM supplemented with 10% FBS, 100 U/ml penicillin, and 0.1 mg/ml streptomycin^25^. Human neuroblastoma SH-SY5Y cells were maintained in RPMI 1640 medium that contained 10% FBS, 100 U/ml penicillin, and 0.1 mg/ml streptomycin^4^. n-propyl gallate (nPG), desferoxamine (DFX), and the anti-β-actin antibody (Ab) were obtained from Sigma (St. Louis, MO, USA). Purified mouse anti-human HIF-1α Ab Clone 54/HIF-1α was purchased from BD Biosciences (San Jose, CA, USA). Anti-HIF-2α (NB100-122) Ab was from Novus Biologicals (Littleton, CO, USA) and HIF-1β/ARNT (D28F3) XP rabbit mAb was from Cell Signaling Technology (Danvers, MA, USA).

### Cell growth assay (MTS assay)

Cell growth was assessed using a CellTiter 96^TM^ AQueous One Solution Cell Proliferation Assay (Promega, Madison, WI, USA)^4^. Briefly, cells were cultured overnight in 96-well plates (2 × 10^4^ cells/well) and treated with the reagents as indicated in the legend to each figure. Twenty μl of CellTiter 96 AQueous One Solution was added to each well, and after 1 hour incubation the absorbance at 490 nm was recorded using an iMark^TM^ Microplate Reader (BIO-RAD, Hercules, CA, USA). All samples were tested 3 or 4 times for each experiment.

### Caspase assays

Caspase-3/7 and caspase-9 activities were assessed using an Apo-ONE^TM^ Homogeneous Caspase-3/7 Assay Kit (Promega) and a Caspase-Glo^TM^ 9 Assays Kit (Promega), respectively, following the manufacturer’s instruction^4^. Briefly, cells were plated in 96-well plates (2 × 10^4^ cells/well) and incubated overnight. Cells were then treated with the appropriate drug(s) for the indicated periods, and caspase activity was measured by adding 100 μl of Apo-One assay reagent to each well. The fluorescence of each sample was measured using an EnSpire^TM^ Multimode Plate Reader (PerkinElmer, Waltham, MA, USA). Assays were performed in triplicate and repeated at least twice. Data were expressed as mean ± standard deviation (SD).

### Immunoblot assays

Whole-cell lysates were prepared as described previously^42–44^ using ice-cold lysis buffer (0.1% SDS, 1% Nonidet P-40 [NP-40], 5 mM EDTA, 150 mM NaCl, 50 mM Tris-Cl [pH 8.0], 2 mM DTT, 1 mM sodium orthovanadate, and Complete Protease Inhibitor^TM^ [Roche Diagnostics, Tokyo, Japan]). Samples were centrifuged at 10,000 × *g* to sediment the cell debris, and the supernatant was used for subsequent immunoblotting experiments. For HIF-α and HIF-1β determinations, 100 µg of protein was fractionated by sodium dodecyl sulfate-polyacrylamide gel electrophoresis (7.5% gel), transferred to membranes and immunoblotted using primary antibodies at a dilution of 1:1,000. Horseradish peroxidase-conjugated to sheep anti-mouse IgG (GE Healthcare, Piscataway, NJ, USA) was used as a secondary antibody at a dilution of 1:1,000. The signal was developed using enhanced chemiluminescence reagent (GE Healthcare). Experiments were repeated at least two times and representative blots are shown.

### Analysis of cell death

Apoptotic cell death was determined using Annexin V-FITC Apoptosis Detection Kit (BioVision, Milpitas, CA, USA), as described previously^4^. Briefly, cells were plated in 6-well plates (3 × 10^5^ cells/well) the day prior to the assay. After overnight incubation, cells were treated with the indicated drug(s) for varying lengths of time and harvested by centrifugation at 260 × *g* for 3 min. The resulting cell pellets were resuspended in 500 μl binding buffer containing 5 μl of Annexin V-FITC and 5 μl of propidium iodide (PI; 50 μg/ml), and stained for 5 min at 25 °C in the dark. Cells were analyzed using a FACSCalibur flow cytometer (BD Biosciences) equipped with CellQuest Pro^TM^ software. Data analysis was performed using FlowJo^TM^ version 7.6.3 software (TreeStar, Ashland, OR, USA), and the results were exported to Excel spreadsheets, subsequently analyzed with the statistical application Prism7^TM^.

### Lactate dehydrogenase (LDH)-based cytotoxic assay

Cell cytotoxicity was measured using CytoTox-ONE^TM^ Kit (Promega) as described previoulsy^4^. Briefly, cells were cultured overnight in 96-well plates (2 × 10^4^ cells/well) and treated with the indicated drug(s) for varying lengths of time. Twenty μl of CytoTox-ONE^TM^ reagent was added to each well and plates were incubated at 22 °C for 10 min. The reaction was terminated by adding 50 µl of Stop Solution, and the fluorescence was recorded with an excitation wavelength of 560 nm and an emission wavelength of 590 nm using an EnSpire^TM^ Multimode Plate Reader (PerkinElmer). The percentage of cell death was determined by comparing the release of LDH (fluorescence value) from each treatment group with that of the positive control treated with Lysis solution, which was defined as 100%. Meanwhile, the value for LDH release from untreated cells (negative control) was defined as 0%. Each sample was assayed in triplicate.

### Determination of mitochondrial membrane potential (ΔΨm)

Mitochondrial membrane potential was assessed by flow cytometry with MitoPT^TM^ JC-1 Assay Kit (ImmunoChemistry Technologies, Bloomington, MN, USA), according to the manufacturer’s instructions^4^. Cells were seeded in 6-well plates (3 × 10^5^ cells/well) and cultured overnight. After treatment with the appropriate drug(s), cells were collected by centrifugation at 260 × *g* for 3 min. Cell pellets were resuspended in JC-1 staining solution and incubated at 37 °C for 15 min in the dark. Samples were subsequently analyzed using a FACSCalibur flow cytometer (BD Biosciences) equipped with CellQuest Pro^TM^ software for detecting JC-1 aggregates with red fluorescence (590 nm emission) or the monomeric form (green fluorescence; 527 nm emission). The data were evaluated using FlowJo version 7.6.3 software (TreeStar, San Carlos, CA, USA), and the results were exported to Excel spreadsheets, subsequently analyzed using the statistical application Prims7^TM^.

### ATP assay

The CellTiter-Glo^TM^ luminescent cell viability assay Kit (Promega, Madison, WI) was used to evaluate the intracellular ATP content. Cells were seeded in 96-well plates. After 24 hours of attachment to the bottom of the plates, 50 μl of the CellTiter-Glo reagent was added directly into each well for a 10-minute incubation. The plate was read by an EnSpire^TM^ Multimode Plate Reader (PerkinElmer, Waltham, MA, USA) after incubation to monitor the luminescence signal generated by the luciferase-catalyzed reaction of luciferin and ATP. Assays were performed in triplicate at least twice. ATP content was then calculated by comparing the luminescence levels of RCC4-VHL cells with that of RCC4-EV cells, which was defined as 100%. Data were expressed as mean ± standard deviation (SD).

### Gene silencing by siRNA

Cells were grown until 30–50% confluence prior to plating on a 24-well plate using DMEM without antibiotics^44^. The cells were then transfected with Validated Stealth RNAi (100 pmol/mL) for HIF-1α (sense: 5′-GGAUGCUGGUGAUUUGGAUAUUGAAdTdT-3′, antisense: 5′-UUCAAUAUCCAAAUCACCAGCAUCCdTdT-3′), HIF-2α (sense: 5′-CAGCAUCUUUGAUAGCAGUdTdT-3′ and antisense: 5′-ACUGCUAUCAAAGAUGCUGdTdT-3), or with the Stealth RNAi Negative Control Kit (Invitrogen Corp., Carlsbad, CA, USA) using Lipofectamine RNAiMAX (Invitrogen Corp.), according to the manufacturer’s instructions. Transfected cells were incubated in a normoxic incubator for 24 h following siRNA transfection.

### Hypoxic treatment

Cells were maintained in a multigas incubator (APMW-36; Astec, Fukuoka, Japan) and exposed to hypoxic conditions (1% O_2_–5% CO_2_–94% N_2_) for the indicated times^42^.

### Cellular oxygen consumption and extracellular acidification measurement

Cellular oxygen consumption rate (OCR) and extracellular acidification rate (ECAR) were detected using the XF Cell Mito Stress Test^TM^ and XF Glycolysis Stress Test^TM^ by an Extracellular Flux Analyzer^TM^ XFp respectively (Agilent Technologies, Santa Clara, CA). RCC4 and RCC4-VHL cells (2 × 10^5^) were seeded onto each well of an XFp Cell Culture microplate. OCR was assessed in glucose-containing XF base medium according to the manufacturer’s instructions. The sensor cartridge for the XFp analyzer was hydrated in a 37 °C non-CO_2_ incubator a day before experiment. For the OCR assay, the sensor cartridge was loaded with 1.5 μM oligomycin (complex V inhibitor) into injection port A, 2 μM FCCP into port B, and 0.5 μM rotenone/antimycin A (inhibitors of complex I and complex III) into port C. During the sensor calibration, cells were incubated in the 37 °C non-CO_2_ incubator in 180 μl assay medium (XF Base Medium, 5.5 mM glucose, 1 mM pyruvate, and 2 mM l-glutamine, pH 7.4). The plate was immediately placed onto the calibrated XFp Extracellular Flux Analyzer for the Mito Stress Test (Supplementary Fig. 4). Calculations were as follows:

OCR (basal) = OCR (last rate measurement before oligomycin injection) − OCR (minimum rate measurement after rotenone/antimycin-A injection).

OCR (maximal) = OCR (maximum rate measurement after FCCP injection) − OCR (minimum rate measurement after rotenone/antimycin-A injection).

OCR (non-mitochondrial respiration) = OCR (minimum rate measurement after Rotenone/antimycin-A injection)

Proton Leak = OCR (minimum rate measurement after Oligomycin injection) − OCR (non-mitochondrial respiration).

For the ECAR assay, the injection port A on the sensor cartridge was loaded with 10 mM glucose. During the sensor calibration, cells were incubated in the 37 °C non-CO_2_ incubator in 180 μl assay medium (XF Base Medium and 2 mM l-glutamine, pH 7.4). The plate was immediately placed into the calibrated XFp Extracellular Flux Analyzer for the Glycolysis Stress Test. Oligomycin (1 µM) and 2-DG (50 mM) were used for the measurement. ECAR was normalized to the total protein/well. ECAR (Glycolysis) = ECAR (maximum rate measurement after glucose injection) − ECAR (last rate measurement before glucose injection).

### Quantitative RT-PCR analysis (*q*RT-PCR)

Total RNA was extracted from cells using the TaKaRa FastPure RNA kit (Takara Bio, Ohtsu, Japan), according to the manufacturer’s instructions. First-strand synthesis and RT-PCR were performed using the One-step SYBR PrimeScript RT-PCR kit (Takara Bio), according to the manufacturer’s protocol. Amplification and detection were performed using the Applied Biosystems 7300 Real-time PCR System (Applied Biosystems, Foster City, CA, USA). PCR primers were purchased from Qiagen. The change in expression of each target mRNA was calculated relative to the level of 18 S rRNA^44^.

### Data analysis of RNA-Seq

FASTQ files for RCC4-EV cells (SRR1554431 and SRR1554986) and RCC4-VHL cells (SRR1554988 and SRR155499) were obtained from the Sequence Read Archive (https://trace.ddbj.nig.ac.jp/dra/index_e.html). The quality of sequence data was evaluated by FastQC (http://www.bioinformatics.babraham.ac.uk/projects/fastqc/) after the trimming process by fastx_toolkit v 0.0.14 (http://hannonlab.cshl.edu/fastx_toolkit/). The human reference sequence file (hs37d5.fa) was downloaded from the 1000 genome ftp site (ftp://ftp.1000genomes.ebi.ac.uk/vol1/ftp/technical/reference/phase2_reference_assembly_sequence/), and the annotated general feature format (gff) file was downloaded from the Illumina iGenome ftp site (ftp://igenome:G3nom3s4u@ussd-ftp.illumina.com/Homo_sapiens/NCBI/build37.2/). The human genome index was constructed with bowtie-build in Bowtie v.2.2.9^45^. The fastq files were aligned to the reference genomic sequence by TopHat v.2.1.1 with default parameters^46^. Bowtie2 v2.2.9 and Samtools v.1.3.1 was used with the TopHat program^47^. Estimation of transcript abundance was calculated, and the count values were normalized to the upper quartile of the fragments per kilobase of transcript per million fragments mapped reads (FPKM) using Cufflinks (cuffdiff) v2.1.1^48^.

Metascape (http://metascape.org/) was used for the gene set enrichment analysis. A gene list for metascape analysis was generated using the output from the cuffdiff program, in which 72 genes judged as ‘significantly differentially expressed’ (p < 0.05) in cuffdiff output (gene_exp. diff, Supplementary Dataset S1) were contained (Supplementary Fig. S3a).

Genes containing Gene Ontology annotations (‘canonical glycolysis’ (GO:0061621) and ‘hypoxia-inducible factor-1alpha signaling pathway’ (GO:0097411)) were extracted using Ensembl Biomart^49^ and sorted by corresponding values (common logarithms of ([FPKM of RCC4-EV] + 1)/([FPKM of RCC4-VHL] + 1)) calculated from the same cuffdiff output file (Supplementary Fig. S3b). The integer one was added to all FPKM values because we cannot calculate the logarithm of 0. The histogram was generated using TIBCO Spotfire Desktop v7.6.0 with TIBCO Spotfire’s “Better World” program license (TIBCO Spotfire, Inc., Palo Alto, CA, USA) (http://spotfire.tibco.com/better-world-donation-program/).

### Statistical Analysis

All experiments were repeated at least twice and each sample was evaluated in triplicate. Representative data, expressed as mean ± SD, are shown. Differences between results were evaluated by one-way analysis of variance (ANOVA) or two-way ANOVA, followed by Dunnet’s test for multiple comparisons or by the t-test using Prism7^Tm^. *P*-values < 0.05 were considered statistically significant.

## Electronic supplementary material

Supplementary Information

Supplementary Dataset S1
