# Supplementary material for: HIF-1-mediated suppression of mitochondria electron transport chain function confers resistance to lidocaine-induced cell death
Source: Sci Rep. 2017 Jun 19;7:3816. doi: 10.1038/s41598-017-03980-7 (PMC5476559; doi:10.1038/s41598-017-03980-7)
Supplement: Supplementary file 1 — Supplementary Information [file 41598_2017_3980_MOESM1_ESM.doc]

**Supplementary Information**

HIF-1-mediated suppression of mitochondria electron transport chain function confers resistance to lidocaine-induced cell death

Authors:

Akihisa Okamoto^1, 2^, Chisato Sumi^1, 2^, Hiromasa Tanaka ^2^, Munenori Kusunoki^1, 2^, Teppei Iwai ^1^, Kenichiro Nishi ^1^, Yoshiyuki Matsuo ^2^, Hiroshi Harada^3, 4^, Keizo Takenaga ^5^, Hidemasa Bono ^6^ and Kiichi Hirota ^2^ *

Affiliations:

^1^ Department of Anesthesiology, Kansai Medical University, Hirakata, Japan, ^2^ Department of Human Stress Response Science, Institute of Biomedical Science, Kansai Medical University, Hirakata, Japan, ^3^ Laboratory of Cancer Cell Biology, Radiation Biology Center, Kyoto University, Kyoto, Japan, ^4^ Precursory Research for Embryonic Science and Technology (PRESTO), Japan Science and Technology Agency (JST), Saitama, Japan, ^5^ Department of Life Science, Shimane University Facluty of Medicine, Izumo, Japan, ^6^ Database Center for Life Science (DBCLS), Research Organization of Information and Systems (ROIS), Mishima, Japan.

**Supplementary Figure 1**

**
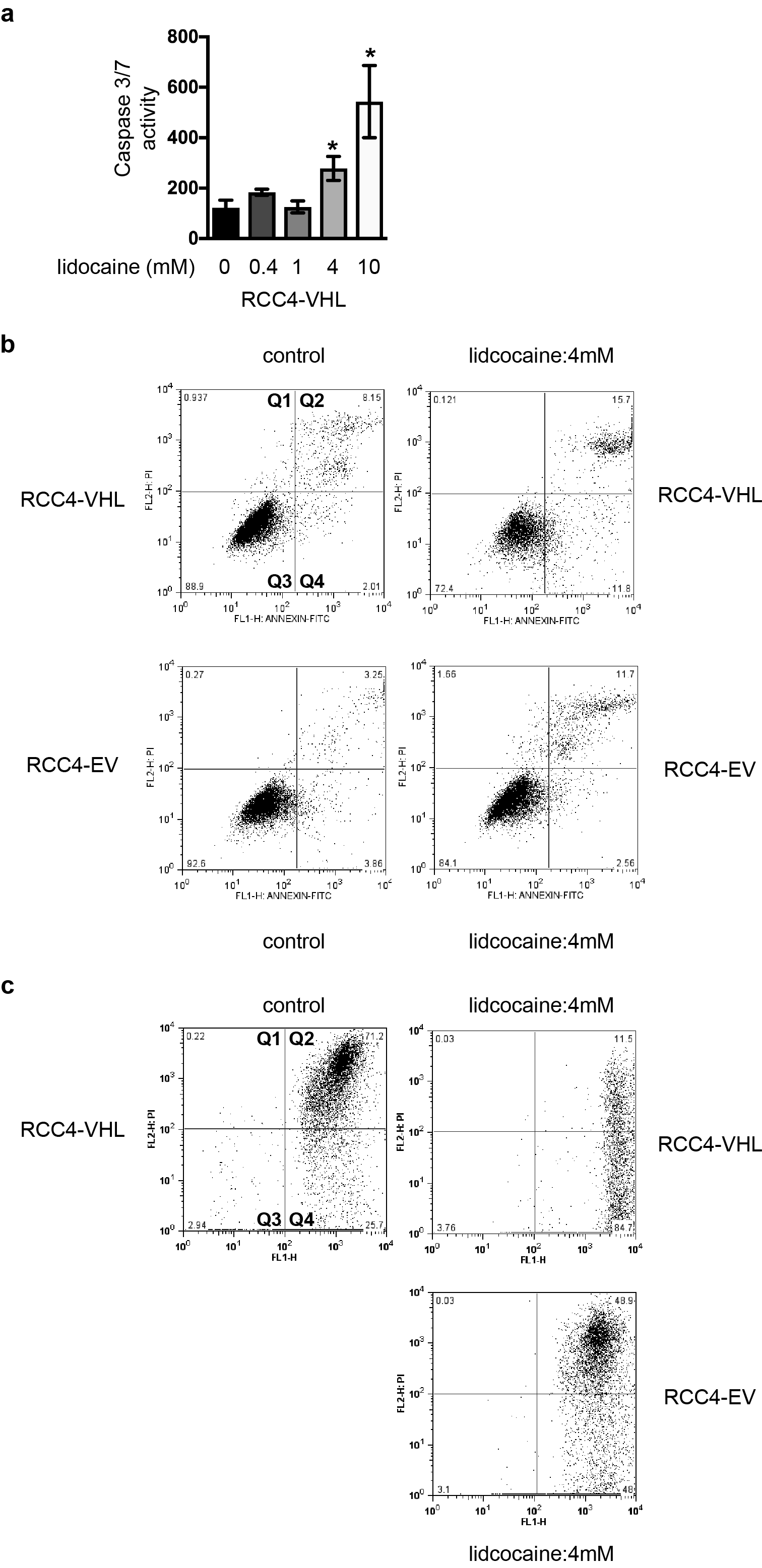
**

Supplementary Figure 1 | Experimental results from caspase 3/7 activation by lidocaine treatment and FACS analyses of cell death

(a) Lidocaine dose-response relationship for caspase 3/7 activation in RCC4-VHL cells

RCC4-VHLwere exposed to the indicated concentrations (0.4, 1, 4, or 10 mM) of lidocaine for 12h. Graphic depictions of caspase-3/7 (n = 5) in each treatment group at different time points

Data presented are expressed as mean ± standard deviation (SD). Differences between results were evaluated by one-way ANOVA followed by Dunnett’s test for multiple comparisons. **p* < 0.05 compared to the control cell population at incubation time 0 h (no treatment).

(b) Analysis of cell apoptosis by FACS

Levels of cell apoptosis were measured using an Annexin V-FITC Apoptosis Detection Kit (BioVision, Milpitas, CA, USA), according to the manufacturer’s instructions. For these analyses, RCC4 cells were seeded into 6-well plates (3 × 10^5^ cells/well) and incubated overnight. The following day, cells were treated with 4 mM lidocaine for 12 h and harvested by centrifugation at 260 xg for 3 min. The culture supernatants were discarded and the resulting pellets were resuspended in 500 μl binding buffer, 5 μl Annexin V-FITC, and 5 μl propidium iodide (PI; 50 μg/ml) for 5 min at 25°C in the dark and analyzed using a FACSCalibur flow cytometer (BD Biosciences, San Jose, CA, USA).

(c) Mitochondrial membrane potential (ΔΨm)

Mitochondrial membrane potential was determined by flow cytometry using a MitoPT™ JC-1 Assay Kit (ImmunoChemistry Technologies, Bloomington, MN, USA), according to the manufacturer’s instructions. For these analyses, RCC4 cells were seeded into 6-well plates (3 × 10^5^ cells/well) and cultured overnight. The following day, cells were treated with 4 mM lidocaine for 4 h and then pelleted by centrifugation at 260 xg for 3 min. Supernatants were discarded and cells were resuspended in JC-1, incubated at 37 °C for 15 min in the dark, and collected by centrifugation at 260 xg for 3 min. Supernatants were again discarded and the remaining cell residues were suspended in 500 μl assay buffer. Samples were subsequently analyzed using a FACSCalibur flow cytometer (BD Biosciences) equipped with CellQuest Pro™ software for the detection of red JC-1 aggregates (590 nm emission) or green JC-1 monomers (527 nm emission).

**Supplementary Figure 2**

**
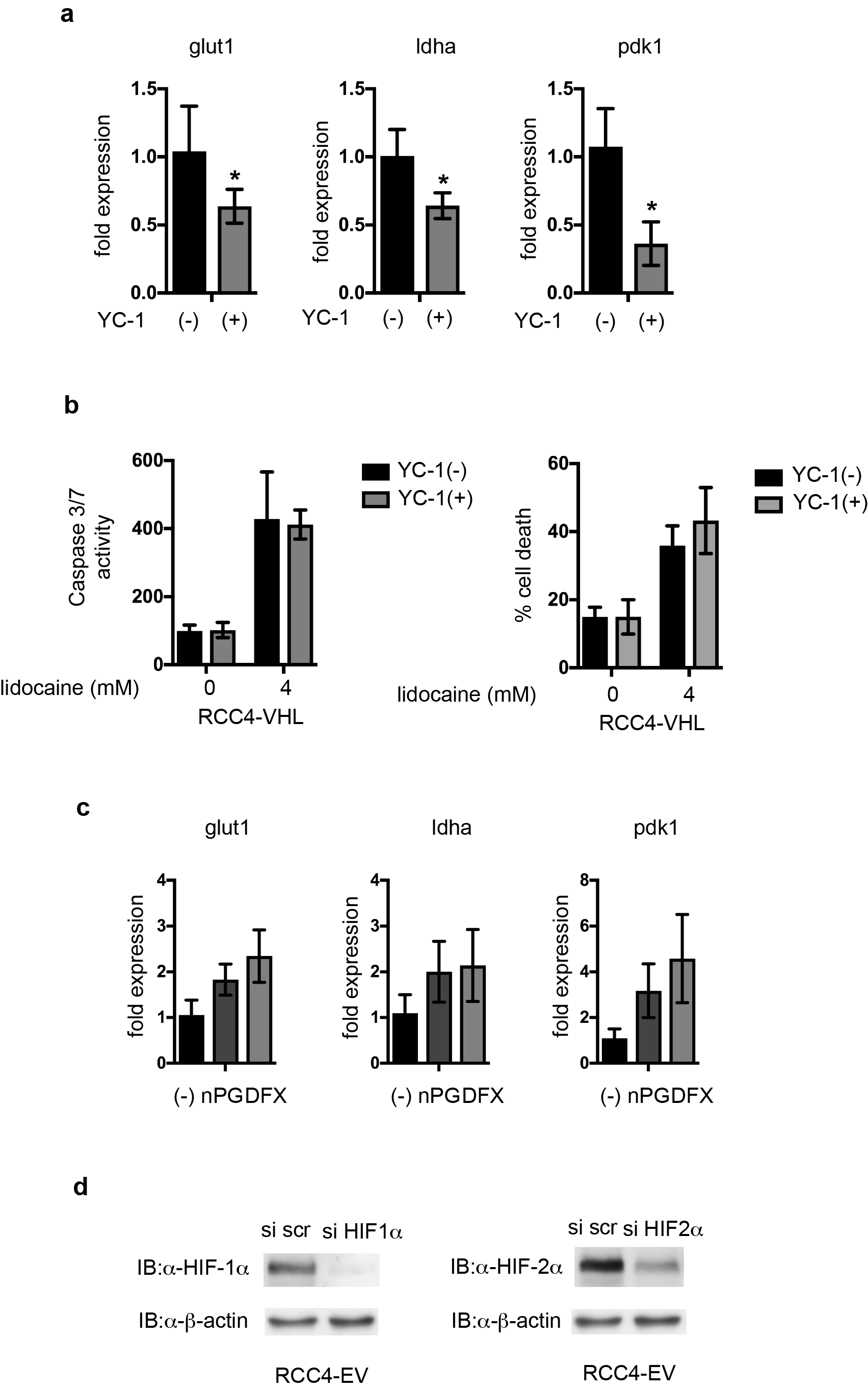
**

Supplementary Figure 2 | Expression of HIF-1-downstream genes in RCC4 cells

(a) RCC4-EV cells were cultured for 6 h in 20 % O_2_ with or without YC-1 to analyze mRNA expression of glucose transporter 1 (glut1), lactate dehydrogenase A (lhda), and pyruvate dehydrogenase kinase 1 (pdk1) using the quantitative real-time reverse transcriptase polymerase chain reaction (*q*RT-PCR). Fold expression was calculated relative to the signal in RCC4-EV cells without YC-1 treatment.

(b) RCC4-VHL cells were cultured for 6 h in 20 % O_2_ with or without treatment of 100 µM nPG and 130 µM DFX to analyze mRNA expression of *glut1*, *lhda*, and *pdk1* using *q*RT-PCR. Fold expression was calculated relative to RCC4-VHL cells without treatment.

(c) RCC4-VHL cells were exposed to 4 mM of lidocaine for 24 h with or without 100 µM of YC-1. (c, left panel) Graphic depiction of caspase-3/7 activity (n = 5). (c, right panel) Cells were harvested and cell death percentages were analyzed by flow cytometry. **p* < 0.05 compared to the non-treated cell population.

(d) RCC4-EV cells were transfected with small interfering RNA (siRNA) targeting HIF-1α (hif1a), HIF-2α (hif2a), or a negative control (scr). Whole-cell lysates were immunoblotted (IB) using anti-HIF-1α, HIF-2α and β-actin antibodies.

**Supplementary Figure 3**

**
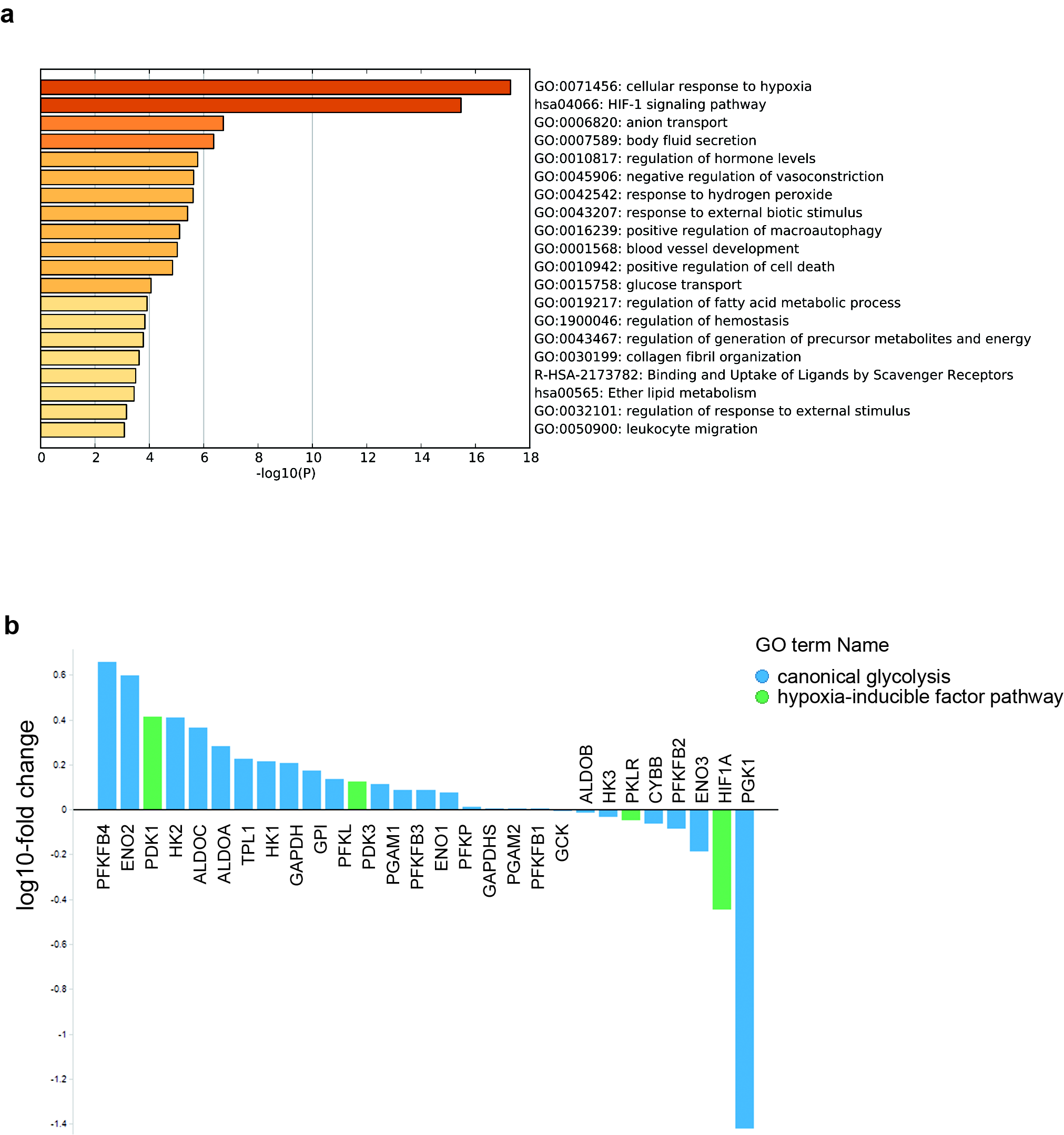
**

Supplementary Figure 3 | Results of gene set enrichment analysis

(a) Heatmap of enriched terms across input gene lists, colored by p-values. Metascape (<http://metascape.org/>) was used for the gene set enrichment analysis. A gene list for metascape analysis was generated using the output from the cuffdiff program, in which 72 genes judged as ‘significantly differentially expressed’ in cuffdiff output (gene_exp.diff) were contained.

(b) RNA-seq of expression levels of selected genes based on GO Term Name (accession GO:0061621 and GO:0097411) in RCC4-EV cells and RCC4-VHL cells. Y axis: ratio of the average value of fragments per kilobase of transcript per million mapped reads (FPKM) for RCC4-VHL cells.

**Supplementary Figure 4**

**
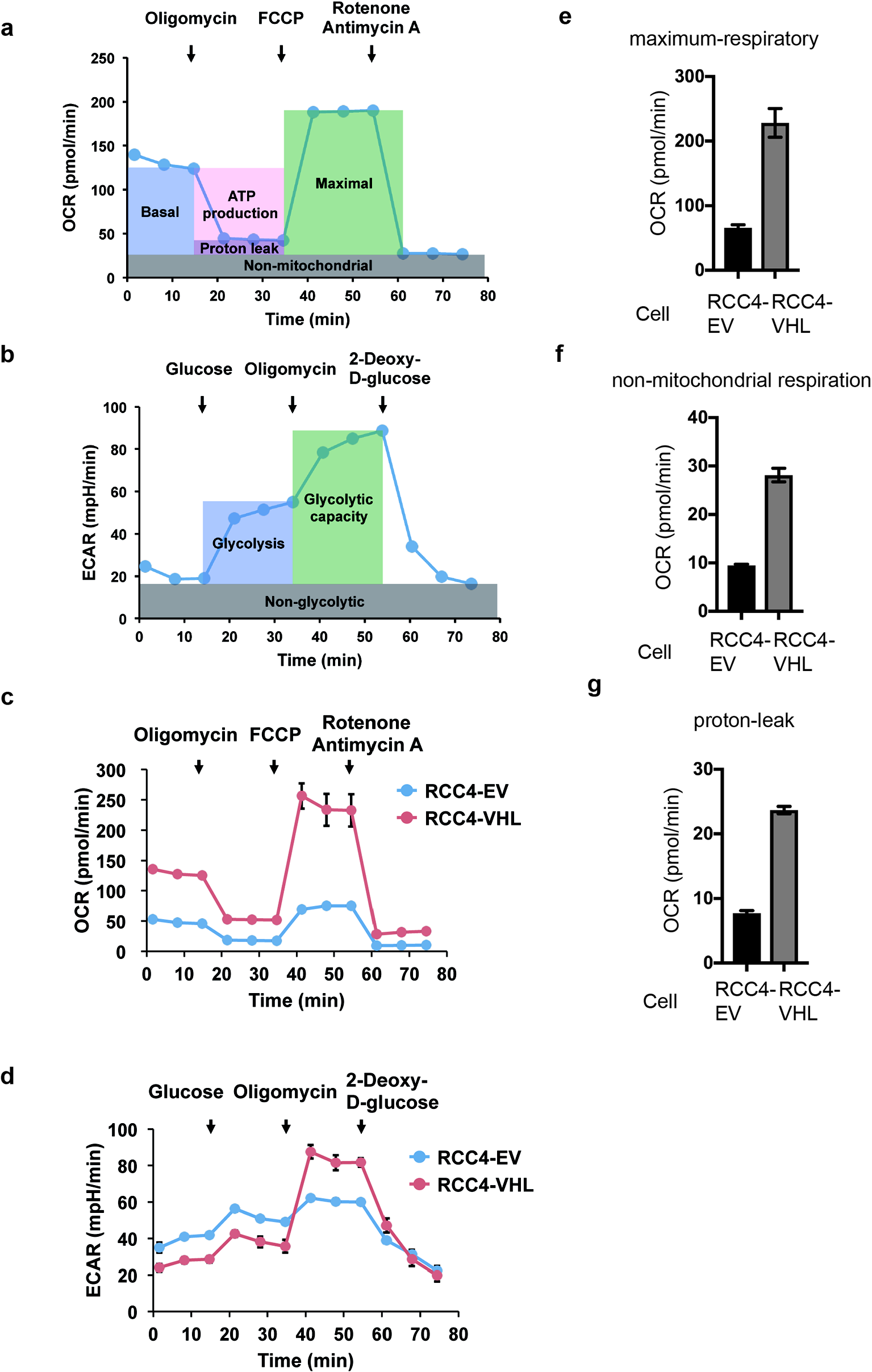
**

Supplementary Figure 4 | Demonstration of OCR and ECAR measurement

(a) Cell Mito Stress test profile of the key parameters of mitochondrial oxygen consumption rate (OCR)

Sequential compound injections measure basal respiration, proton leak, maximal respiration, and non-mitochondrial respiration.

(b) Cell Glycolysis Stress test profile of the key parameters in extracellular acidification rate (ECAR)

(c) OCRs in RCC4-VHL and RCC4-EV cells. Oligomycin (Oligo), FCCP, and antimycin A/rotenone (Anti/Rot) were injected at the indicated time points. Data presented as mean ± SD.

(d) ECAR in RCC4-VHL and RCC4-EV cells. Data presented as mean ± SD.

OCR (maximal)(e), OCR (non-mitochondrial respiration)(f) and Proton Leak(g) in RCC4-EV cells and RCC4-VHL cells were demonstrated.

**Supplementary Figure 5**

**
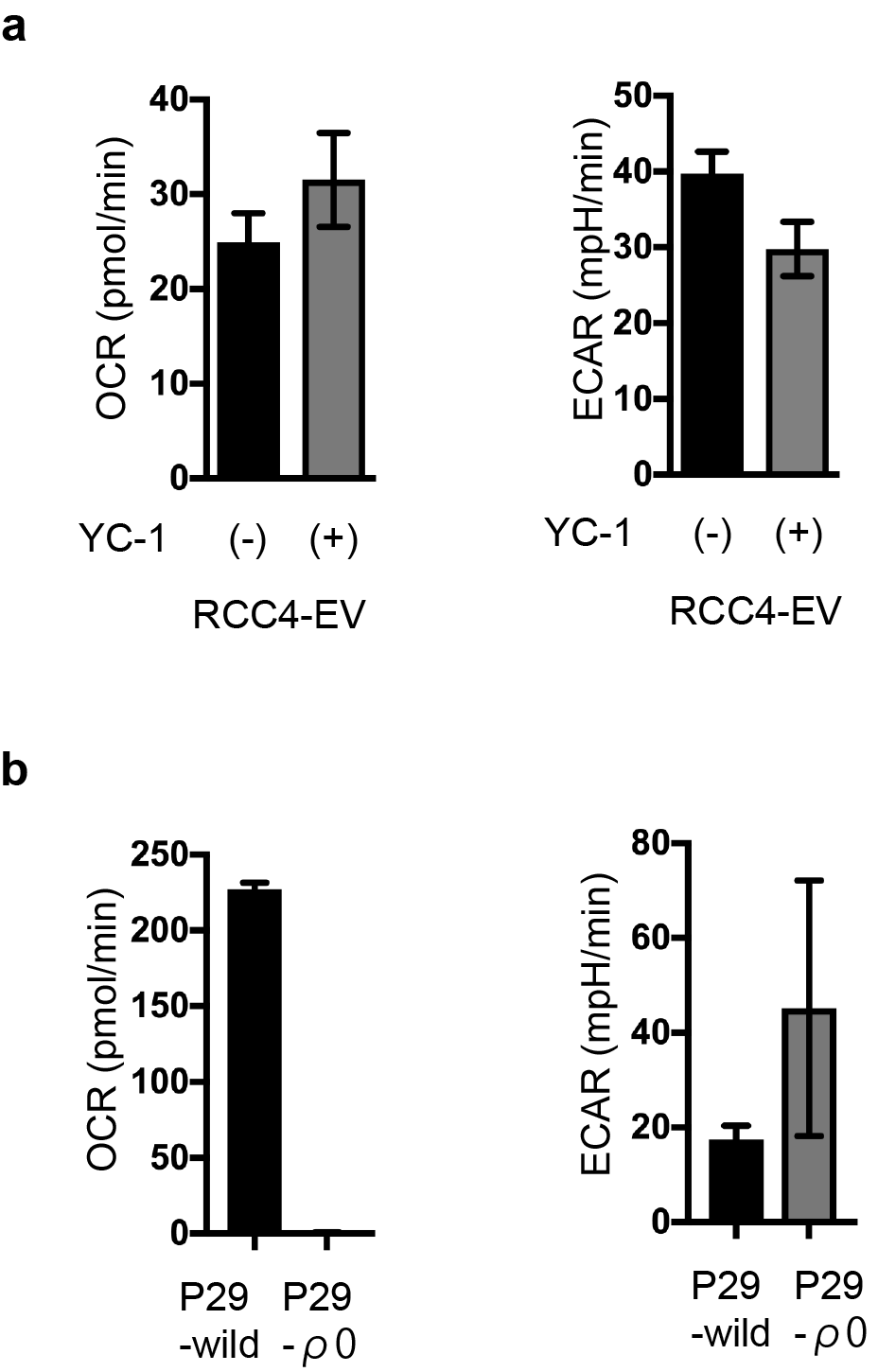
**

Supplementary Figure S5 | Results of OCR and ECAR assays in RCC4 cells and P29 cells

(a) Oxygen consumption rate (OCR) (right panel) and extracellular acidification rate (ECAR) (left panel) for RCC4-EV cells with or without YC-1 treatment (n=3).

(b) Oxygen consumption rate (OCR) (right panel) and extracellular acidification rate (ECAR) (left panel) of P29 wild cells and P29 ρ0 cells (n=3).

**Supplementary Figure 6**

**
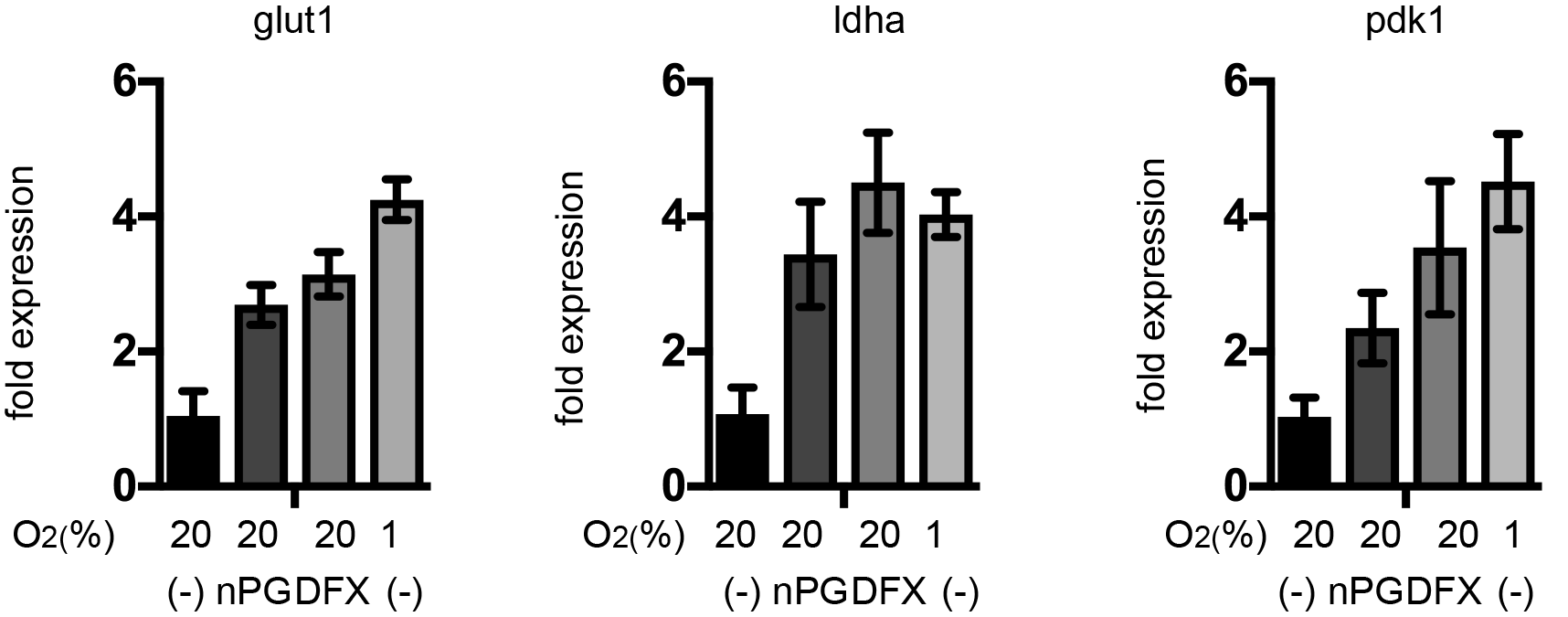
**

Supplementary Figure 6 | HIF-1-downstream gene expression in SH-SY5Y cells

SH-SY5Y cells were treated with 100 µM nPG or 130 µM DFX for 6 h. Analysis of glucose transporter 1(glut1), lactate dehydrogenase A (lhda), and pyruvate dehydrogenase kinase 1 (pdk1) mRNA levels using real-time quantitative reverse transcriptase polymerase chain reaction (*q*RT-PCR). Fold expression was calculated relative to RCC4-VHL cells in 20 % O_2_.
